# Modeling Spatial Patterns of Soil Respiration in Maize Fields from Vegetation and Soil Property Factors with the Use of Remote Sensing and Geographical Information System

**DOI:** 10.1371/journal.pone.0105150

**Published:** 2014-08-26

**Authors:** Ni Huang, Li Wang, Yiqiang Guo, Pengyu Hao, Zheng Niu

**Affiliations:** 1 The State Key Laboratory of Remote Sensing Science, Institute of Remote Sensing and Digital Earth, Chinese Academy of Sciences, Beijing, China; 2 Land Consolidation and Rehabilitation Center, Ministry of Land and Resources, Beijing, China; DOE Pacific Northwest National Laboratory, United States of America

## Abstract

To examine the method for estimating the spatial patterns of soil respiration (*R_s_*) in agricultural ecosystems using remote sensing and geographical information system (GIS), *R_s_* rates were measured at 53 sites during the peak growing season of maize in three counties in North China. Through Pearson's correlation analysis, leaf area index (LAI), canopy chlorophyll content, aboveground biomass, soil organic carbon (SOC) content, and soil total nitrogen content were selected as the factors that affected spatial variability in *R_s_* during the peak growing season of maize. The use of a structural equation modeling approach revealed that only LAI and SOC content directly affected *R_s_*. Meanwhile, other factors indirectly affected *R_s_* through LAI and SOC content. When three greenness vegetation indices were extracted from an optical image of an environmental and disaster mitigation satellite in China, enhanced vegetation index (EVI) showed the best correlation with LAI and was thus used as a proxy for LAI to estimate *R_s_* at the regional scale. The spatial distribution of SOC content was obtained by extrapolating the SOC content at the plot scale based on the kriging interpolation method in GIS. When data were pooled for 38 plots, a first-order exponential analysis indicated that approximately 73% of the spatial variability in *R_s_* during the peak growing season of maize can be explained by EVI and SOC content. Further test analysis based on independent data from 15 plots showed that the simple exponential model had acceptable accuracy in estimating the spatial patterns of *R_s_* in maize fields on the basis of remotely sensed EVI and GIS-interpolated SOC content, with R^2^ of 0.69 and root-mean-square error of 0.51 µmol CO_2_ m^−2^ s^−1^. The conclusions from this study provide valuable information for estimates of *R_s_* during the peak growing season of maize in three counties in North China.

## Introduction

Soil CO_2_ efflux from terrestrial ecosystems to the atmosphere has been considered the second largest global carbon flux and is a vital component of ecosystem respiration [1]. In recent decades, significant progress has been made in identifying the biophysical factors that influence soil respiration (*R_s_*) to predict soil CO_2_ emission accurately in time and space [2]–[4].

The majority of *R_s_* arises from root and microbial tissue. Therefore, understanding the spatial and temporal changes of these sources will facilitate the modeling of *R_s_*. However, the large spatial and temporal heterogeneity of root and microbial activity within the landscape and the covariation of potentially important factors (i.e., temperature and water content) pose great challenges to the development of mechanistically based models that account for spatial and temporal variability in *R_s_* [2]. Thus, many different statistical models of *R_s_* have been developed on the basis of data collected from different ecosystems [5]. Numerous studies have established *R_s_* models based on soil temperature, soil moisture, or both [6], [7]. Aside from soil temperature and moisture, plant productivity proxies [e.g., leaf area index (LAI), canopy chlorophyll content (Chl_canopy_), and plant biomass] [8]–[10] and soil properties [e.g., soil organic carbon (SOC) content, soil total nitrogen (STN) content, and soil C and N ratio (soil C/N)] [11], [12] also potentially influence *R_s_* and are often included in models of *R_s_*. However, most of the factors that affect variations in *R_s_* tend to be derived through field measurements [13]. Furthermore, direct observation of these variables across long time spans or large spatial scales is expensive because of the required manpower and material resources. A simple method to derive data related to variations in *R_s_* is necessary to facilitate the determination of the spatial and temporal distribution of *R_s_*.

Remote sensing and geographical information system (GIS) provide powerful tools for data acquisition, spatial analysis, and graphical display [14]–[16]. In the field of global change research, significant advances have been made in the development and application of remote sensing and GIS. These advances include land cover and land-use changes [17], [18], environmental vulnerability and risk assessment [19], [20], ecological restoration and management [21]–[23], and terrestrial ecosystem carbon cycle [24]–[26]. However, applying the data derived from remote sensing and GIS into *R_s_* modeling remains controversial, especially for remote sensing data, because remotely sensed data in principle are independent measurements of site properties, not functionally important variables (e.g., soil temperature, soil moisture, and plant growth variables) that control *R_s_* [3], [27], [28]. On the basis of statistical analysis of field experiments, previous studies found that remotely sensed vegetation indices (VIs) correlate with *R_s_* in crop sites that lack drought stress [10] and can be used to model the spatial patterns of *R_s_* during the peak growing season of alpine grasslands in the Tibetan Plateau [26]. However, few studies explore the potential of remote sensing and GIS data for estimating the spatial patterns of *R_s_* in agricultural land, which may be affected by more complex factors than natural grasslands because of the influence of human activity. Although modern agriculture has successfully increased food production, the processes involved have profoundly affected the global carbon cycle through tillage, drainage and conversion of natural to agricultural ecosystems [29], [30]. Therefore, a simple method should be identified to study the spatial characteristics of *R_s_* in agricultural ecosystems.

This study aims to examine a potential new approach for estimating the spatial patterns of *R_s_* during the peak growing season of maize by using remote sensing and GIS technology in Baixiang, Longyao and Julu Counties, which are typical agricultural areas in the north plain of China. Studying the spatial characteristics of soil CO_2_ efflux in maize fields will contribute to eco-agricultural development.

## Materials and Methods

### Ethics Statement

**Figure 1:**
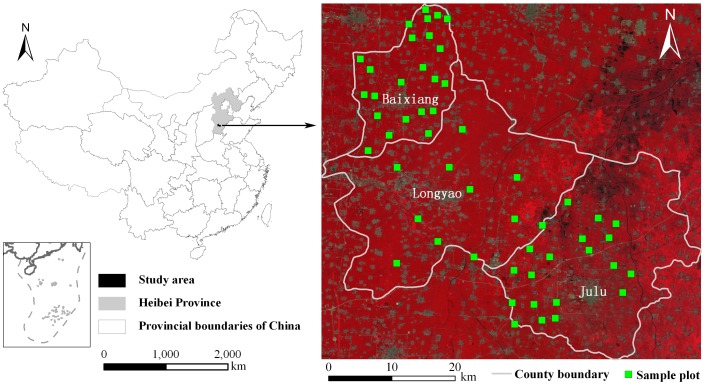
Spatial location of the sample plots for field experiments in three counties in North China. The box in the bottom left corner of Figure 1 shows the South China Sea islands.

No specific permissions were required for the 53 sample plots in this study. We confirmed that the field studies did not involve endangered or protected species, and the specific location of the sample plots was provided in the manuscript (Fig. 1).

### Study Site

The study site is situated within three counties (Baixiang, Longyao and Julu) in Southern Hebei Province of North China (Fig. 1). The total area of the study site is 1.64×10^3^ km^2^. This area is located in the North China Plain with a flat open terrain, single landform type, and a mean elevation of 30 m above sea level. Calcareous alluvial soil with high capacity to retain water and fertilizer is the main soil type in the study area. The study site is suitable for farming, and maize is the main crop. The climate is continental monsoon with four distinct seasons and adequate light and heat resources. Long records of meteorological data near the study site (http://cdc.cma.gov.cn) indicate that the mean annual temperature is 13.5°C with the coldest temperatures in January and the hottest in July. The mean annual precipitation is 502.8 mm, but precipitation is distributed unevenly in the four seasons with the greatest precipitation occurring in summer (362.5 mm). Therefore, drought influences agricultural development, and agriculture mainly involves irrigation in this study site.

Fifty-three sample plots located in the maize fields were identified within the study site (Fig. 1). The distance between any two sample plots was larger than 2 km. Each sample plot (greater than 100 m×100 m) has a large maize area, flat terrain, and maize under uniform growing conditions. All measurements were performed from August 11, 2013 to August 20, 2013, which corresponded to the tassel stage and peak growing period of maize. During the 10 days of field measurements, continuous measurements were performed, except on August 12 because of a minor precipitation event. Therefore, all field measurements required 9 days.

### Field measurements

#### Soil respiration measurements

In each sample plot, *R_s_* was measured by using a soil respiration chamber (LI-6400-09; LiCor, Lincoln, Nebraska, USA) connected to a portable photosynthesis system (LI-6400; LiCor, Lincoln, Nebraska, USA). The soil respiration chamber was mounted on a PVC soil collar that was sharpened at the bottom. Each PVC collar (5 cm long, 11 cm inside diameter) was inserted 2 cm to 3 cm into the ground and was installed at least 24 h prior to performing any measurements. To reduce the difference in root biomass, soil collars were placed in three locations on the basis of their distance to the maize plant: near a maize plant, inter-plant, and inter-row. Two collars were placed in each of the three positions for each *R_s_* measurement. At least three to four consecutive measurements on each collar were performed to prevent any systematic error in the *R_s_* estimates. An average *R_s_* value was used for each collar, and the average value from six collars was used to represent the *R_s_* value at plot level. Each *R_s_* measurement was conducted between 09:00 h and 15:00 h (local time) because fluxes measured during this time interval are usually representative of the daily mean flux.

#### Soil temperature and soil moisture measurements

After the soil respiration measurement on a PVC soil collar in each plot, soil temperature and soil moisture were measured in this collar to minimize sample difference. Soil temperature was measured at a 10 cm depth (T_s10_) by using a ground thermometer. Volumetric soil moisture at a depth of 0 cm to 20 cm (SM_20_) was determined by using a portable time domain reflectometry probe (HydroSense, Campbell, USA). Thus, six soil temperature and moisture measurements were performed in each plot. The average value was used to represent soil temperature or soil moisture at the plot level.

#### Maize biophysical parameter measurements

LAI was measured by using an LAI-2000 (LI-COR Inc., Lincoln, Nebraska). In each plot, six representative positions were selected for LAI measurement, and in every position, two repeated measurements were performed. Leaf chlorophyll content (Chl_leaf_) was determined by using a portable chlorophyll meter (SPAD-502, New Jersey, USA). Fully expanded leaves, which depended on the height of the maize plant, were randomly selected from three locations that corresponded to the upper, middle, and lower parts of the maize plant. For each leaf location, 10 SPAD values were randomly collected. The vertical leaf area distribution in maize canopy was analyzed by measuring the area of each green leaf from the bottom to the top of eight randomly distributed maize plants with the use of an area meter (LI-3100, LI-COR, Lincoln, Nebraska). The area-weighted mean SPAD reading was used to derive Chl_leaf_. However, the SPAD reading was in arbitrary units rather than in actual amounts of chlorophyll per unit area of the leaf tissue. A transform relationship exists between the SPAD readings and the actual chlorophyll content in maize [31]. To convert the SPAD readings to chlorophyll content per unit leaf area (µg cm^−2^), this study used the transform relationship (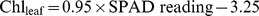) derived by Wu et al. [32] in maize plots, and the same SPAD meter was employed in this study. Chl_canopy_ was then determined by using the following equation:
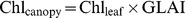
(1)where Chl_canopy_ is the canopy chlorophyll content (g m^−2^), Chl_leaf_ is the leaf chlorophyll content of maize (g m^−2^), and GLAI represents the green leaf area per unit ground area.

In each sample plot, three representative maize plants were harvested for aboveground biomass (AGB) measurement. These fresh maize plants were sealed in a plastic bag and immediately transported to a nearby laboratory for subsequent analysis. Thereafter, fresh samples were oven dried at 65°C until the mass of the sample reached a constant weight. The AGB in each plot can be derived by multiplying the average dry weight per plant (g plant^−1^) and the average plant density of maize (plants m^−2^).

#### Soil property measurements

Soil within the six PVC collars in each plot was destructively sampled after measuring *R_s_*, soil temperature and soil moisture. Soil was sampled to a depth of approximately 20 cm by a cylindrical soil driller (4 cm diameter, 20 cm height), in which fine root biomass and microbial activity are the highest [33], [34]. These collected soil samples were sealed in plastic bags and stored at room temperature while being transported to the laboratory. Six collected soil samples in each plot were uniformly mixed to form a composite sample for laboratory analysis. The composite sample was air-dried in the laboratory to a constant weight for soil chemical analyses. The air-dried soil samples were ground to pass through a 0.2 mm sieve after any visible plant tissues and debris were manually removed. The SOC content was estimated by using the standard Mebius method [35]. The STN content was analyzed by using the Kjeldahl digestion procedure [36]. In this study, soil C/N was calculated by the ratio of SOC and STN contents.

### Spatial data acquisition

#### Maize classification data

This study aimed to derive the spatial distribution of *R_s_* in maize fields based on the field measurements at the plot scale. Maize classification data is necessary to spatially extrapolate *R_s_* at the plot scale to the whole study area. Multi-temporal normalized difference vegetation index (NDVI) data collected over the growing season were used to classify maize at the study site [37]–[39]. Clouds are common occurrences in the study area during the growing season. Thus, obtaining a time sequence of cloud-free scenes is difficult. Two types of satellite data were used to establish the time-series NDVI data. One was the Operational Land Imager (OLI) image of Landsat 8, and the other was the small constellation for environmental and disaster mitigation (HJ-1A and B) charge coupled device (CCD) image [40]–[42]. Five scenes of OLI images acquired on May 3, 2013, May 19, 2013, July 6, 2013, October 10, 2013, and October 26, 2013 were downloaded from the U.S. Geological Survey (http://earthexplorer.usgs.gov/). Three HJ-1A and B CCD optical images acquired on June 6, 2013, August 17, 2013, and September 15, 2013 were downloaded from the China Center for Resource Satellite Data and Applications (http://www.cresda.com). The two types of remote sensing images exhibit same spatial resolution (30 m). The 30 m spatial resolution is appropriate for classifying maize patterns in the study area given the relatively large field in the region, which could spatially corresponded to five or more 30 m pixels. The strong relationship of the NDVI with biophysical vegetation characteristics, such as LAI and green biomass [43], [44], enables the discrimination of land cover types on the basis of their unique phenological responses. Before land-use classification, pre-processing (i.e., radiometric calibration, atmospheric correction and geometric correction) of OLI images and HJ-1A and B CCD optical images was accomplished by using the Environment for Visualizing Images (ENVI) software (Version 4.7, Research Systems Inc., Boulder, Colorado, USA) [45], [46]. This process ensured the consistency between the two types of remote sensing data and the seasonality of the NDVI time series. The maximum likelihood classification method, integrated in the ENVI software, was applied to the eight-date NDVI time series that spanned one maize growing season of the study site.

#### Spectral vegetation index for vegetation biophysical parameter estimation

Three greenness indices, namely, NDVI, enhanced vegetation index (EVI), and modified soil adjusted vegetation index (MSAVI), were derived from the HJ-1A CCD optical image acquired on August 17, 2013 (Table 1) for vegetation biophysical parameter estimation. Previous studies reported that greenness VIs offer important and convenient measures for vegetation biophysical parameters, such as LAI and Chl_canopy_ [51]–[54]. Meanwhile, LAI and Chl_canopy_ are also found to be good indicators of plant canopy photosynthesis [55]–[57] and are used in the modeling of *R_s_* [58]. To obtain the spatial patterns of vegetation biophysical parameters in maize fields, the spatial distribution of vegetation biophysical parameters over the whole study area was overlapped with the maize classification data.

**Table 1:** Calculation for vegetation indices a.

Vegetation index	Formula	Reference
Normalized difference vegetation index	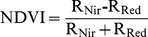	Rouse et al. [47], Gamon et al. [48]
Modified soil adjusted vegetation index	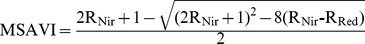	Qi et al. [49]
Enhanced vegetation index	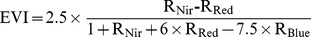	Huete et al. [50]

a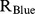, 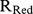, and 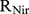 are reflectance of blue, red, and NIR band in the HJ-1A CCD optical image, respectively.

#### Quantifying the spatial pattern of SOC content

Statistics and geostatistics have been widely applied to quantify the spatial distribution patterns of SOC at a regional scale [59]–[61]. Based on the theory of regionalized variables, geostatistics provides advanced tools to quantify the spatial features of soil parameters and to conduct spatial interpolation [62], [63]. In this study, geostatistical analyses were performed by using the geostatistical analyst module of ArcGIS software (Version 9.3, 2008) to quantify the spatial pattern of SOC content. To obtain the spatial pattern of the SOC content in the maize fields, the spatial distribution of the SOC content over the whole study area was overlapped with the maize classification data.

### Modeling spatial patterns of soil respiration

#### Identifying factors affecting spatial variability of soil respiration

The variables that explain the spatial variability of *R_s_* are as follows: (1) soil properties, measured by SOC content, STN content and soil C/N; (2) environmental factors, encompassing T_s10_ and SM_20_, and (3) plant photosynthesis proxy factors, including AGB, LAI and Chl_canopy_. Pearson's correlation requires variables to be normally distributed and mutually independent. Each variable was tested for normal distribution by using the Shapiro–Wilk normality test and for randomness by the runs test of the Statistical Package for the Social Sciences (SPSS, Chicago, Illinois, USA). The results of the statistical analysis showed that each of these measured variables followed a normal distribution (Shapiro-Wilk, p>0.05) and showed randomness (runs test, p>0.05). Thus, Pearson's correlation analysis, as implemented in the SPSS software, was used to screen important variables that influence *R_s_*. Five variables with statistically significant correlation (p<0.05) with *R_s_*, namely, SOC content, STN content, LAI, AGB, and Chl_canopy_, were screened out (Table 2). However, these variables were cross-correlated [64]–[66] and included both direct and indirect effects. To solve this problem, structural equation modeling (SEM) was used to evaluate explicitly the causal relationships among these interacting variables [67]–[69] and to divide the total effects of variables on *R_s_* into direct and indirect effects. On the basis of the theoretical knowledge on the major factors that influence spatial patterns of *R_s_* at regional scales [8], [13], [26], we developed an SEM model to relate *R_s_* to SOC content, STN content, LAI, AGB, and Chl_canopy_. This SEM model was used to identify the direct effect factors for *R_s_* estimation. The SEM model was fitted by using AMOS 18.0 for Windows [70]. After using the SEM, the fit indices, namely, comparative fit index = 0.984 and goodness-of-fit index = 0.946. Thus, the theoretical model showed a good fit with the sample data.

**Table 2:** Pearson's correlation among soil respiration and factors affecting soil respiration in maize fields during the peak growing season in three counties in North China.

	R_s_	T_s10_	SWC_20_	Chl_canopy_	LAI	AGB	SOC content	STN content	Soil C/N
R_s_	1.00	−0.27	−0.18	0.54***	0.75***	0.59***	0.76***	0.59***	−0.23
T_s10_		1.00	0.18	−0.15	−0.28	−0.27	−0.49**	−0.66***	0.51**
SWC_20_			1.00	−0.17	−0.05	−0.07	0.16	−0.00	0.06
Chl_canopy_				1.00	0.83***	0.81***	0.26	0.20	−0.18
LAI					1.00	0.76***	0.44**	0.38*	−0.28
AGB						1.00	0.45**	0.34*	−0.15
SOC content							1.00	0.78***	−0.29
STN content								1.00	−0.79***
Soil C/N									1.00

*R_s_* is the daily mean soil respiration rate (µmol CO_2_ m^−2^ s^−1^), T_s10_ is the soil temperature at 10 cm depth (°C), SWC_20_ is the soil water content at 0 cm to 20 cm depth (m^3^ m^−3^), Chl_canopy_ is the canopy chlorophyll content (g m^−2^), LAI is the leaf area index, AGB is the aboveground biomass (kg m^−2^), SOC content is the soil organic carbon content (g kg^−1^), STN content is the soil total nitrogen content (g kg^−1^), and soil C/N is the soil C∶ N ratio. Significance levels:

*p<0.05,

**p<0.01,

***p<0.001.

#### Quantifying the spatial patterns of soil respiration in maize fields

In this study, the direct effect factors of *R_s_* identified by SEM were used to estimate *R_s_*. The spatial distribution data of these direct effect factors were first obtained on the basis of remote sensing or GIS to quantify the spatial patterns of *R_s_* in maize fields. A simple exponential model that used the proxy data was then employed to estimate the spatial pattern of *R_s_* during the peak growing season of maize. The accuracy of this method was examined by separating the observed data into two datasets through a random generator. One dataset consisted of 38 sample plots for analysis, whereas the other consisted of 15 for testing the accuracy of the *R_s_* estimation.

## Result

### Spatial characteristics of soil respiration

Based on field-measured data at 38 plots, the daily mean *R_s_* of maize during the peak growing season was 5.43 µmol CO_2_ m^−2^ s^−1^ with a range of 2.64 µmol CO_2_ m^−2^ s^−1^ to 7.33 µmol CO_2_ m^−2^ s^−1^ and a coefficient of variation (CV) of 15.45% (Table 3). The spatial variability of soil temperature at 10 cm depth (T_s10_) was relatively small at the study site with a CV of 4.73% and was far less than the spatial variation in soil water content at 0 cm to 20 cm depth (SWC_20_). The AGB of maize showed greater spatial variability (CV = 31.93%) than LAI (CV = 8.64%) and Chl_canopy_ (CV = 6.54%).

**Table 3:** Spatial characteristics of soil respiration (*R_s_*, µmol CO_2_ m^−2^ s^−1^), soil temperature at 10 cm depth (T_s10_, °C), soil water content at 0 cm to 20 cm depth (SWC_20_, m^3^ m^−3^), canopy chlorophyll content (Chl_canopy_, g m^−2^), leaf area index (LAI), aboveground biomass (AGB, kg m^−2^), soil organic carbon content (SOC content, g kg^−1^), soil total nitrogen content (STN content, g kg^−1^) and soil C∶ N ratio (soil C/N) in maize fields during the peak growing season in three counties in North China.

Variables	Mean	Maximum	Minimum	CV (%)
R_s_	5.43	7.33	2.64	15.45
T_s10_	28.32	30.93	25.78	4.73
SWC_20_	27.54	33.27	19.54	12.48
Chl_canopy_	0.18	0.21	0.16	6.54
LAI	3.75	4.53	2.81	8.64
AGB	0.94	1.89	0.44	31.93
SOC content	11.86	17.26	6.40	16.71
STN content	1.25	1.78	0.53	24.47
Soil C/N	9.82	14.38	7.07	18.53

Mean SOC content, STN content, and soil C/N at 0 cm to 20 cm depth in maize fields of the study site were 11.86 g kg^−1^ (ranged from 6.40 g kg^−1^ to 17.26 g kg^−1^), 1.25 g kg^−1^ (ranged from 0.53 g kg^−1^ to 1.78 g kg^−1^), and 9.82 (ranged from 7.07 to 14.38), respectively. Their CVs were not similar with the STN content which showed greater spatial variability than the SOC content and soil C/N.

### Factors driving spatial variability of soil respiration

**Figure 2:**
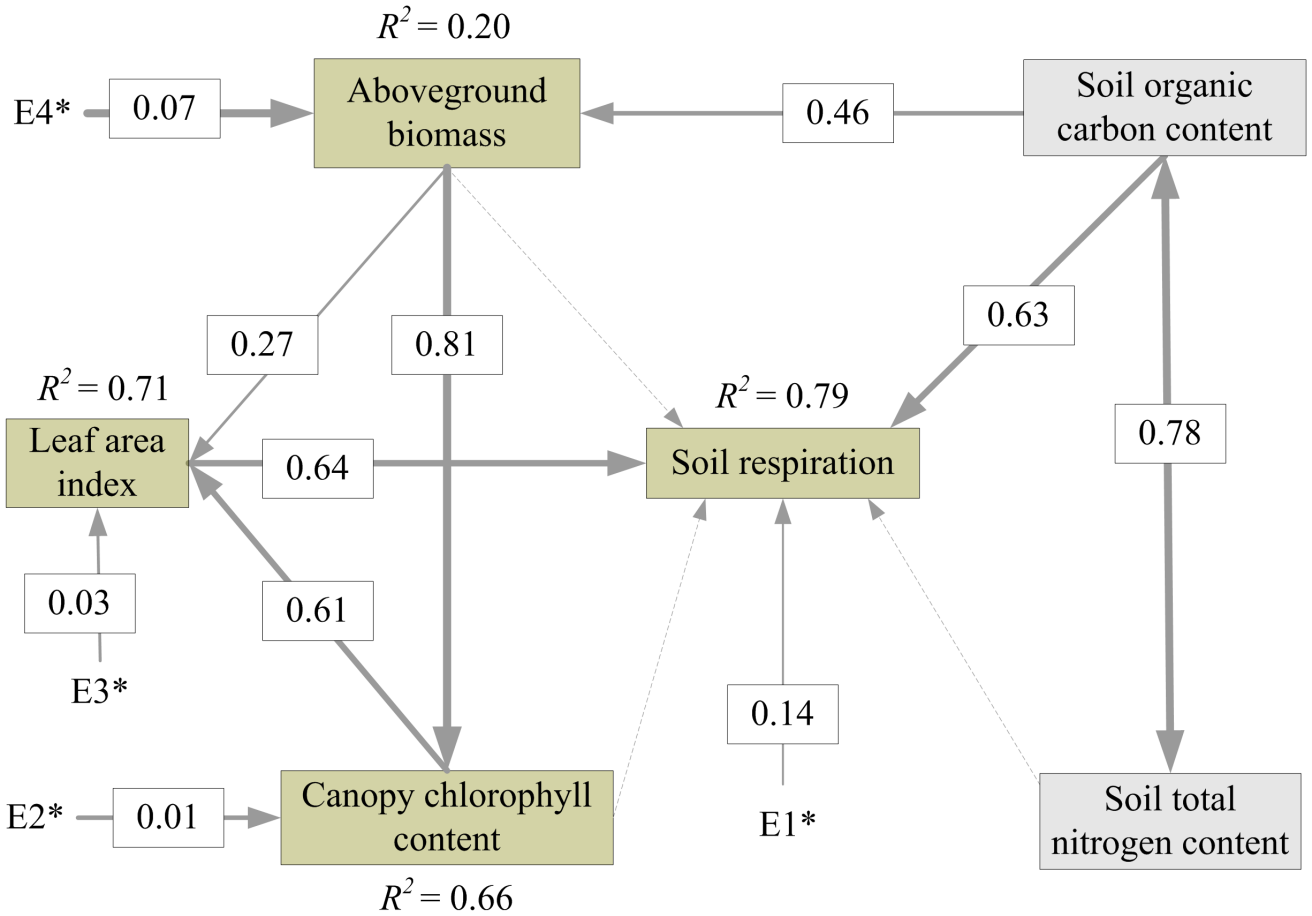
Final structural equation modeling (SEM) for soil respiration. Non-significant paths are shown in dashed line. The thickness of the solid arrows reflects the magnitude of the standardized SEM coefficients. Standardized coefficients are listed on each significant path. * represents error terms for observed variables, among them, E1, E2, E3, and E4 represent measurement errors for soil respiration, canopy chlorophyll content, leaf area index, and aboveground biomass, respectively.

Based on Pearson's correlation analysis, five variables with significant correlation with *R_s_*, namely, Chl_canopy_, LAI, AGB, SOC content, and STN content, were selected (Table 2). However, the five selected variables were intercorrelated (Table 2), and their relationships with *R_s_* combined both direct and indirect correlations. Thus, an SEM model was further used to evaluate the causal relationships among these interacting variables. The final SEM explained 79% of the variation in *R_s_* (Fig. 2). The direct, indirect, and total effects of the variables are shown in Table 4. Among the five selected variables, LAI and SOC content directly affected *R_s_* and can be used to predict *R_s_* with relatively high accuracy (R^2^ = 0.79). The other three variables (i.e., Chl_canopy_, ABG, and STN content), despite having a significant correlation with *R_s_*, only affected *R_s_* indirectly through their direct relationship with SOC content and LAI. Thus, the two direct effect factors were used to estimate *R_s_*, and the spatially distributed data proxies of these two factors were used to quantify the spatial patterns of *R_s_* in maize fields during the peak growing season.

**Table 4:** Total, direct, and indirect effects in the structural equation modeling.

Variable	Direct effect	Indirect effect	Total
**Soil respiration**			
Aboveground biomass	−0.10*ns*	0.46	0.36
Soil organic carbon content	0.63	0.16	0.79
Soil total nitrogen content	−0.09*ns*	0.30	0. 21
Leaf area index	0.64	-	0.64
Canopy chlorophyll content	−0.04*ns*	0.39	0.35
**Aboveground biomass**			
Soil organic carbon content	0.46	-	0.46
Soil total nitrogen content	−0.01*ns*	-	−0.01*ns*
**Leaf area index**			
Aboveground biomass	0.27	0.50	0.77
Soil organic carbon content	-	0.35	0.35
Soil total nitrogen content	-	−0.01*ns*	−0.01*ns*
Canopy chlorophyll content	0.61	-	0.61
**Canopy chlorophyll content**			
Aboveground biomass	0.81	-	0.81
Soil organic carbon content	-	0.37	0.37
Soil total nitrogen content	-	−0.01*ns*	−0.01*ns*

These effects were calculated using standardized path coefficients. Non-significant effects are indicated by “*ns*”.

### Spatial data used for soil respiration estimation

#### Maize classification

**Figure 3:**
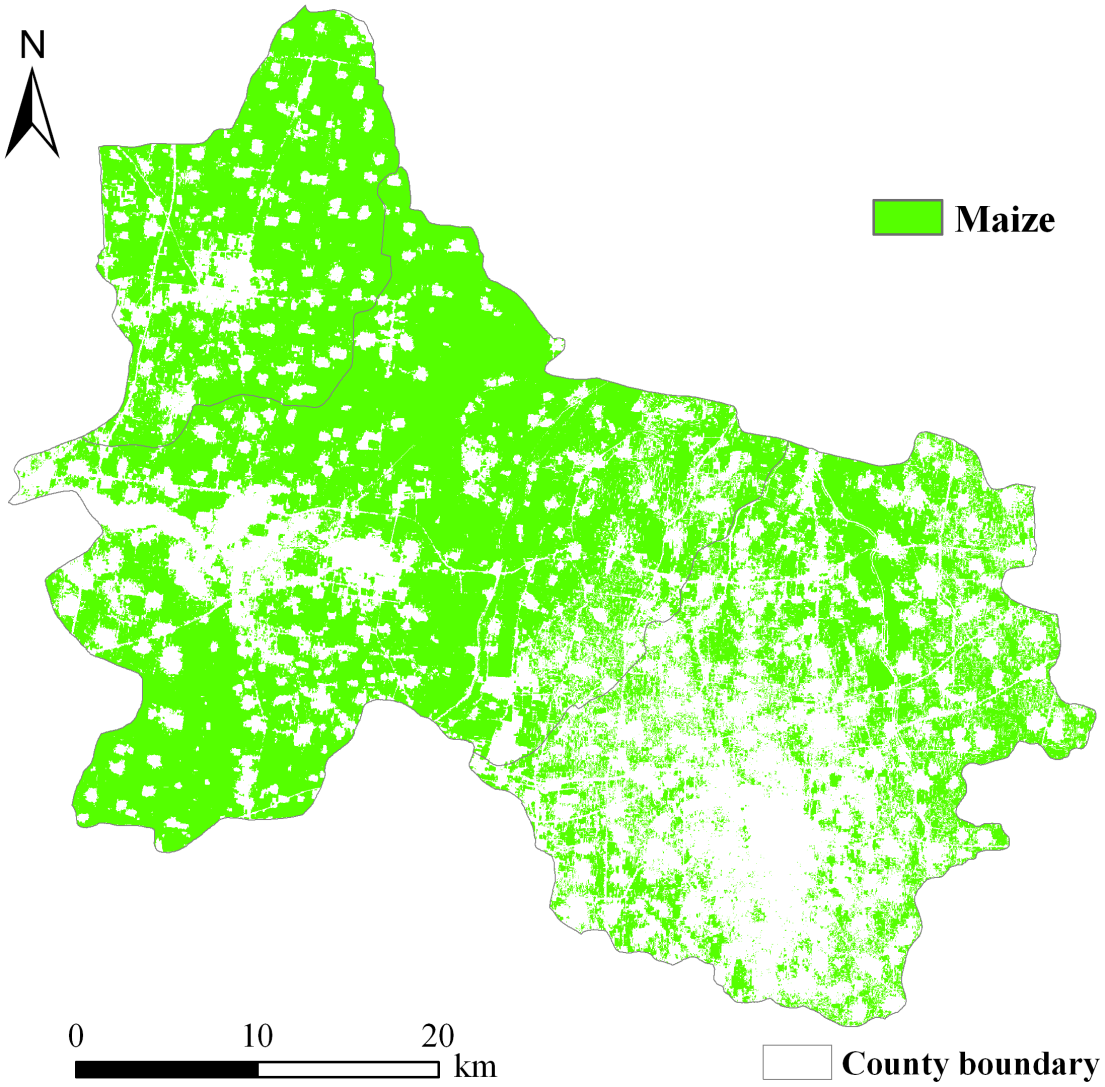
Maize classification map in three counties in North China.

The maize classification map of the study area is shown in Figure 3. The classification accuracy for maize at the study site could not be quantitatively assessed because of the limitation of the sample data. However, 53 sample plots were all located in the maize classification map, and the county-level maize patterns classified in the map were consistent with the general maize patterns across the three counties. In addition, the classified maize area was close to the maize area reported by the China County Statistical Yearbook [71]. Thus, the classification accuracy of maize was believed to be reasonable, and the maize classification map was then used to predict the spatial pattern of *R_s_* during the peak growing season of maize.

#### LAI estimation from spectral vegetation index

**Figure 4:**
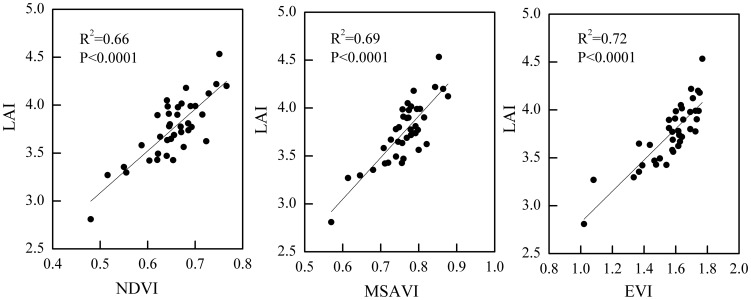
Linear relationships between three vegetation indices (VIs) and leaf area index (LAI) during the peak growing season of maize in three counties in North China (n = 38). The VIs are: normalized difference vegetation index (NDVI), enhanced vegetation index (EVI), and modified soil adjusted vegetation index (MSAVI).

**Figure 5:**
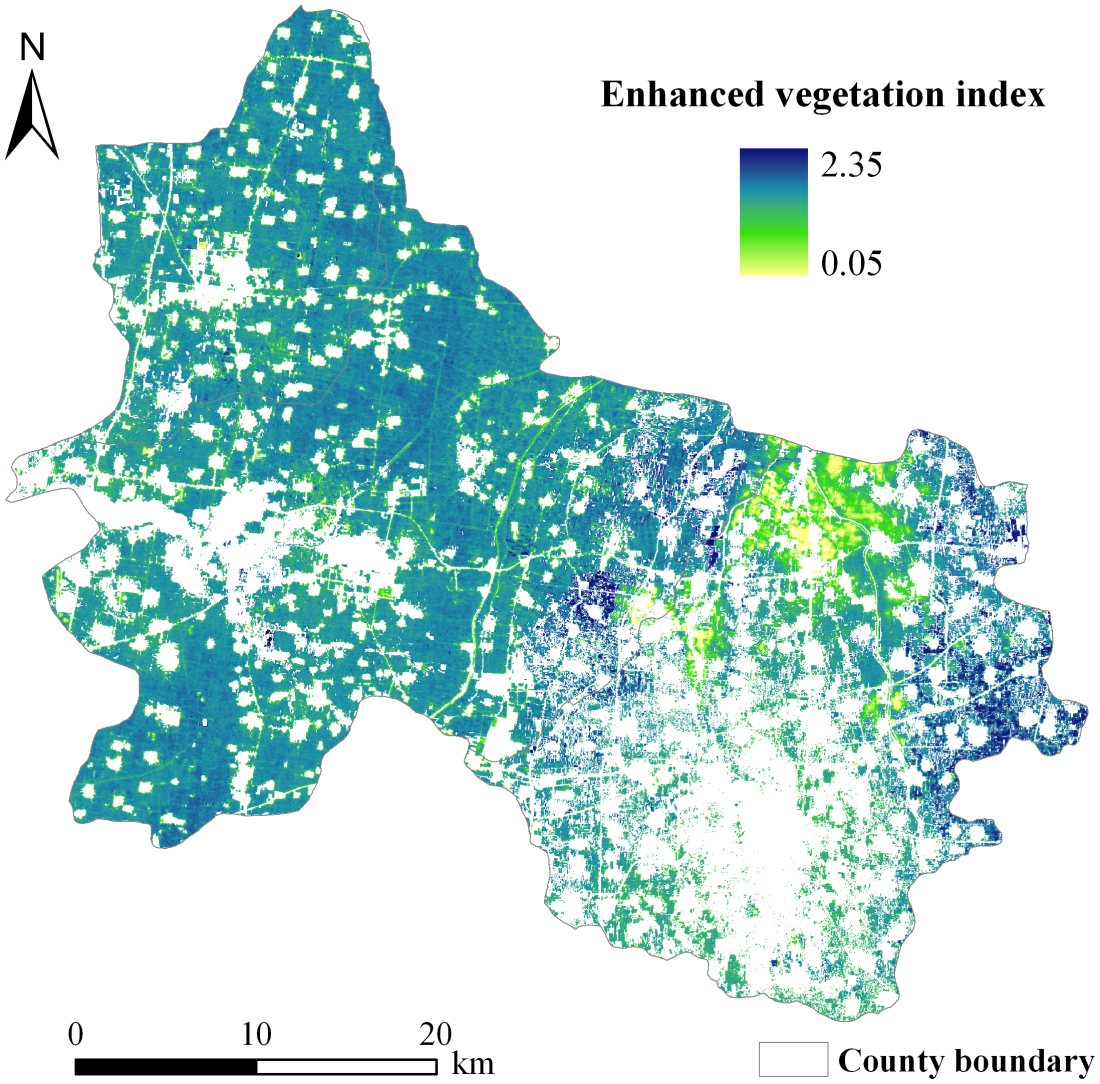
Spatial distribution map of enhanced vegetation index in maize fields in three counties in North China.

Among the three greenness indices calculated from the optical image of HJ-1A satellite, EVI showed the best linear relationship with LAI, with a determination coefficient (R^2^) of 0.72, followed by MSAVI and NDVI (Fig. 4). The explanation of LAI variance increased from 66% to 72% when EVI was used instead of NDVI for LAI estimation, and this increase was statistically significant (p<0.05). However, EVI and MSAVI did not exhibit a significant difference in explaining the variation in LAI, despite EVI having a slightly better relationship with LAI than MSAVI. Thus, EVI was used as a proxy for LAI to estimate *R_s_* during the peak growing season of maize for simplicity. The spatially distributed EVI during the peak growing season of maize exhibited relatively small variability (Fig. 5). Overall, the EVI in the north and southwest parts of the study site (i.e., Baixiang and Longyao Counties) showed a high value. Relatively low EVI values mainly occurred in the southeast parts of the study site (i.e., Julu County), especially the northwest Julu County (Fig. 5).

#### Spatial distribution of SOC content

**Figure 6:**
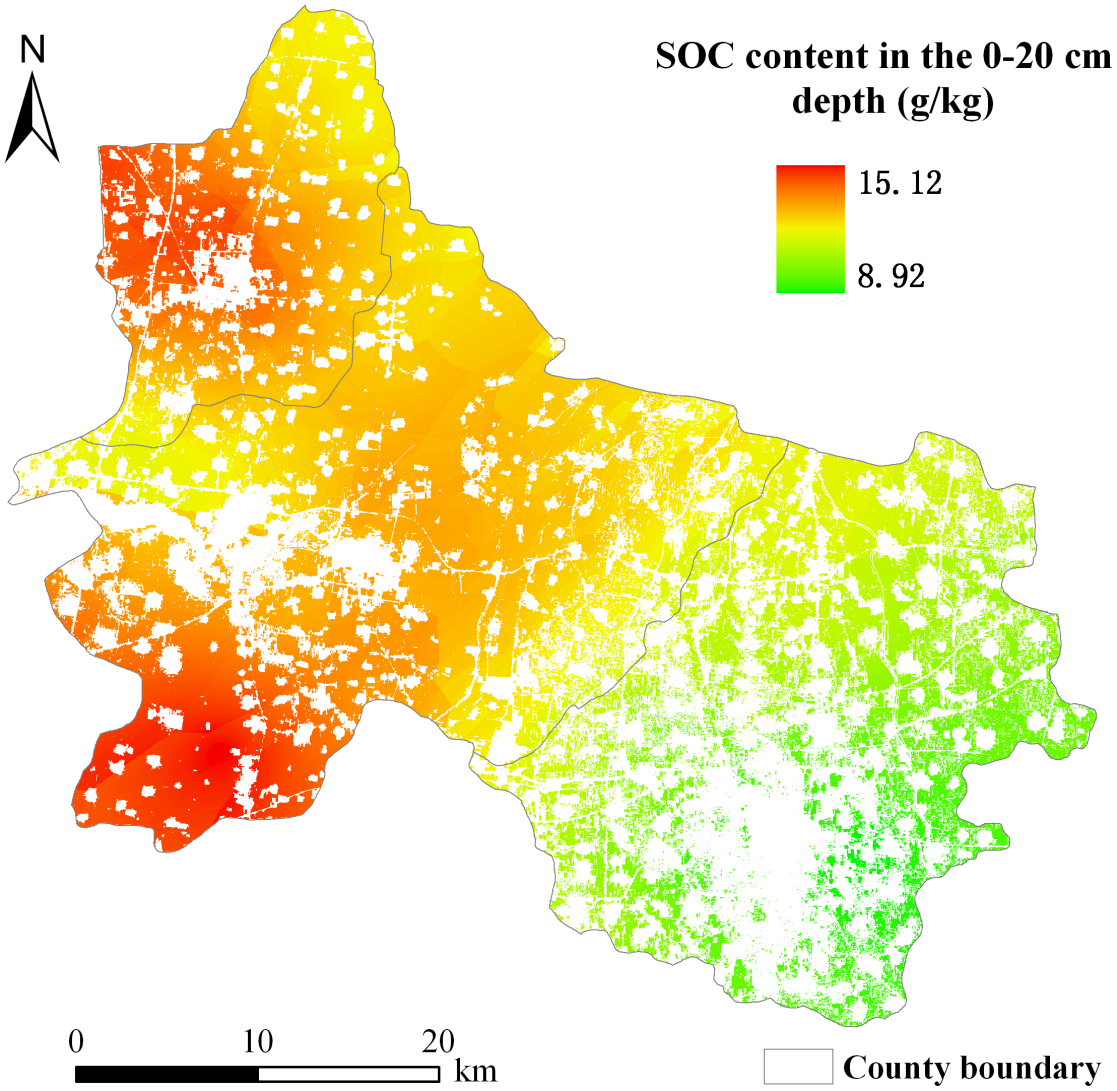
Spatial distribution map of soil organic carbon (SOC) content in the 0–20 cm depth in maize fields in three counties in North China.

Kriging interpolation was performed by using ArcGIS 9.3 software to produce the spatial distribution map of the SOC content in maize fields of the study area. A cell size of 30 m×30 m was selected for the spatial interpolation to match the spatial resolution of images from OLI and HJ-1A/B. The final result of this spatial interpolation process is shown in Figure 6. Based on the spatial distribution map of the SOC content in maize fields, SOC content values were higher in the northwest and southwest parts of the study area than in the southeastern part.

### Spatial distribution of soil respiration

**Figure 7:**
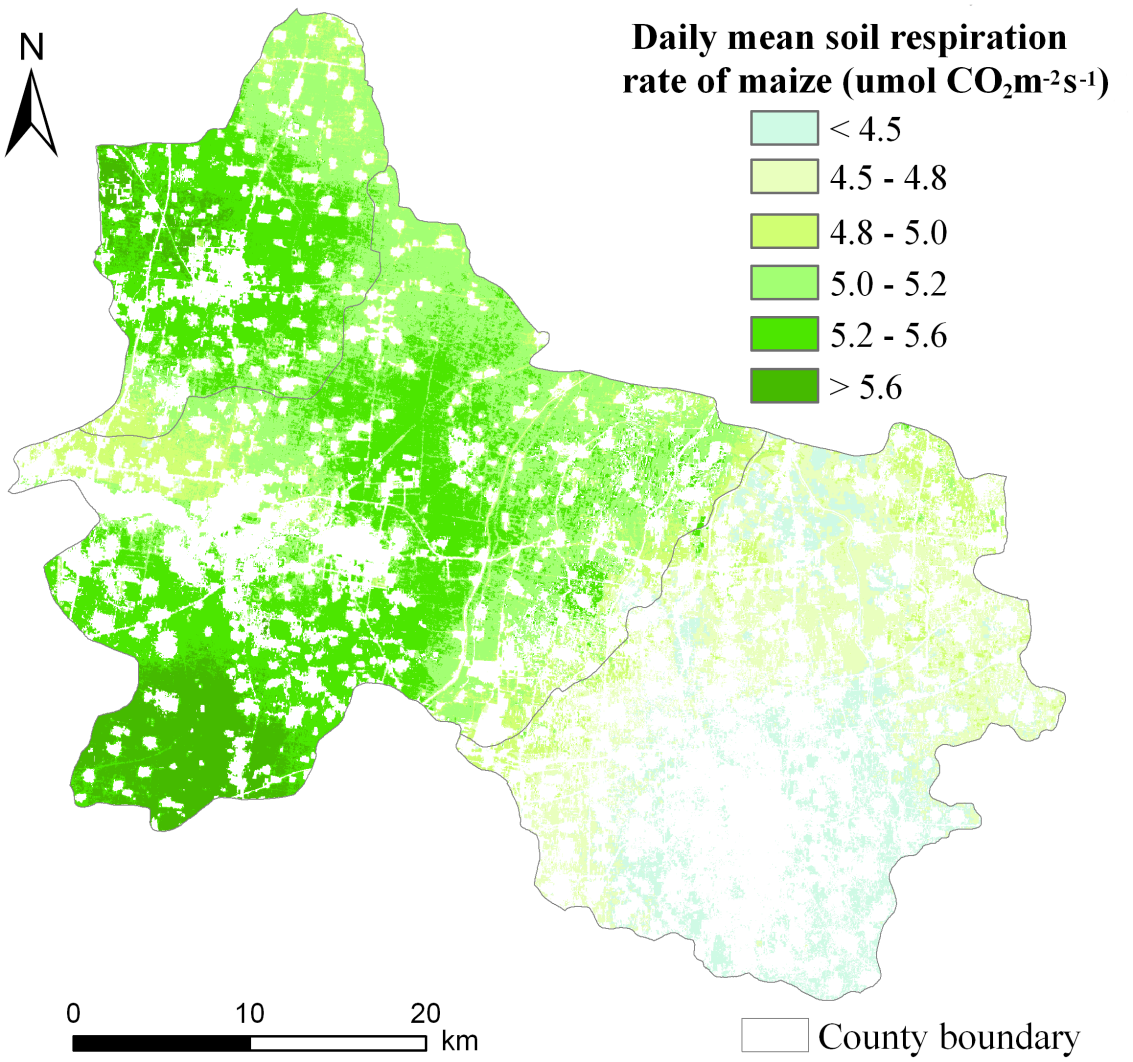
Spatial pattern of daily mean soil respiration rate during the peak growing season of maize in three counties in North China.

The EVI and SOC content were used to estimate the spatial pattern of *R_s_* during the peak growing season of maize on the basis of a simple exponential model. The geo-location information (latitude and longitude) of the 38 sample plots was used in the extraction of pixels. Pixels that contained these plots from the spatial distribution maps of EVI and SOC content data (Figs. 5 and 6) were extracted. These data were used to determine the model parameters by least-squares fitting. The resulting model was as follows:
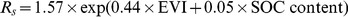
(2)

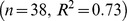
where *R_s_* refers to the daily mean soil respiration rate in µmol CO_2_ m^−2^ s^−1^; EVI refers to enhanced vegetation index, as a proxy for LAI; and SOC content is the soil organic carbon content (g kg^−1^) in maize fields of the study area. Eq. (2) was employed to predict the spatial pattern of *R_s_* from spatially distributed EVI and SOC content data during the peak growing season of maize (Figs. 5 and 6). The spatial variation in *R_s_* showed a pattern similar to that in SOC content (Figs. 6 and 7).

**Figure 8:**
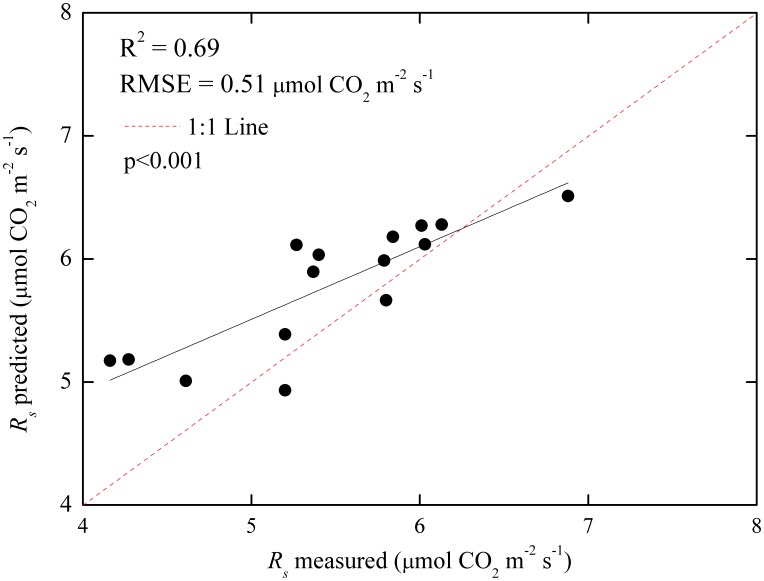
Spatial data predicted soil respiration (*R_s_*) and corresponding ground-based measurements with R^2^ and RMSE (µmol CO_2_ m^−2^ s^−1^) during the peak growing season of maize in three counties in North China (*n* = 15). The predicted soil respiration was attained with an exponential model that used EVI and SOC content as dependent variables.

Figure 8 shows the accuracy assessment result of the *R_s_* prediction model. The field measured *R_s_* was comparable with the spatial data predicted *R_s_*. Based on the independent test dataset, EVI and SOC content accounted for 69% of the spatial variation in ground-measured *R_s_*, and the RMSE was 0.51 µmol CO_2_ m^−2^ s^−1^. The result of the accuracy assessment suggests that the prediction model, which used EVI and SOC content as the dependent variables, was effective in estimating *R_s_* in maize fields during the peak growing season.

## Discussion

### Relationships between LAI and three VIs

In this study, in situ measured data were obtained during the peak growing period of maize (corresponding to the tassel stage of maize). The effect of soil background on the spectral reflectance of remote sensing images was negligible during this period because the maize cover was higher with LAI ranging from 2.81 to 4.53. The difference in the capability of spectral vegetation index (VI) responding to LAI variation mainly depended on the sensitivity of VI to the canopy structural variation of maize. Thus, the VI modified the effect of soil reflectance (i.e. MSAVI) did not exhibit a significantly greater advantage than NDVI, which is strongly affected by soil reflectance in sparsely vegetated areas [50]. EVI, which is more sensitive to variation in dense vegetation than NDVI [50], showed the best relationship with the LAI of maize. This result was consistent with our previous study [58] that was conducted in irrigated and rainfed maize fields located at the University of Nebraska, Agricultural and Research Development Center, Mead, Eastern Nebraska, USA.

### Measurement accuracy of SOC content

Field measurement data revealed that the SOC content at 0 cm to 20 cm depth in the maize fields ranged from 6.4 g kg^−1^ to 17.3 g kg^−1^, and the mean value was 12.01 g kg^−1^. For the mean dry land SOC content in North China, the value appeared to be higher than the previous estimate (0.83 from the average of 268 sample points) [72]. This difference was partly attributed to the fact that only the SOC content in maize fields, not in all dry land types, was considered. Most maize fields in the study site were on a winter wheat/maize rotation, and wheat straw was returned to the soil. The high productivity of maize crops contributed to the development of a thick A horizon and high SOC content [73], [74]. Additionally, only the SOC content in maize fields at 0 cm to 20 cm depth was analyzed, whereas previous studies estimated the SOC content on the basis of organic carbon content to a depth of 1 m [72], [75], [76]. In agricultural land, soil depth at 0 cm to 20 cm is located in the cultivation layer and has a higher SOC content than the SOC content at the deeper soil layers [34]. This condition contributed to the higher SOC content from the measured soil property data than the previous estimate.

### Factors affecting spatial pattern of soil respiration

The spatial differences in *R_s_* at the study site can be mainly attributed to the differences in vegetation productivity and soil property factors among the sample plots, whereas soil temperature and soil moisture served a minor function in regulating the spatial pattern of *R_s_*. A previous study also demonstrated that site variables that reflect site productivity (e.g., LAI or aboveground net primary productivity) will provide a useful approach for large-scale estimates of regional *R_s_* in terrestrial ecosystems [8]. Soil temperature evidently serves a predominant function in the spatial variations of *R_s_* across sites of climatically contrasting environments [4]. However, at a local scale or under similar climatic conditions, other biological and biophysical factors, such as vegetation productivity and the size of organic carbon pools, may prevail as dominant drivers of *R_s_* [4], [77]. At a local scale, the spatial variation in T_s10_ in the study site was small (CV = 4.73%). Thus, soil temperature did not affect the spatial pattern of *R_s_*. Although soil moisture in the maize fields showed a relatively large spatial variation (CV = 12.48%), this variation did not reach a degree that will affect the spatial dynamics of *R_s_*. The soil C quantity and substrate quality factors (i.e., SOC and STN contents) were consistently and strongly correlated with one another and significantly affected the variation in *R_s_* [5], [12], [13]. However, SEM results showed that the STN content only affected *R_s_* indirectly through the direct effect on the SOC content at the study site.

During the peak growing season of maize, biophysical parameters, such as LAI, Chl_canopy_, and AGB, were important variables that determined the size of the photosynthetic capacity [56], [78]. However, these variables are not truly independent, and a correlation between one of them and *R_s_* may lead to a correlation of the other with *R_s_*. In this study, *R_s_* was strongly correlated with LAI, Chl_canopy_ and AGB of maize fields, whereas LAI was the only variable directly related to *R_s_* during the peak growing season of maize on the basis of SEM analysis.

The direct effect factors of *R_s_* were used to estimate the spatial variability of *R_s_* during the peak growing season of maize in three counties in North China. A simple exponential model, which included the corresponding spatial proxies from remote sensing and GIS (i.e., EVI and spatially interpolated SOC content), was employed. A similar method was applied to a deciduous broadleaf forest site in the Midwest USA [79]. The independent test data also demonstrated the rationality of this method at the study site to a certain extent (Fig. 8). Regardless of the form of the *R_s_* model, the relationship between LAI and EVI, as well as the kriging interpolation precision of the SOC content, affected the predictive accuracy of the *R_s_* model. A moderate correlation between EVI and LAI (Fig. 4) affected the test accuracy of the exponential model with an R^2^ value of 0.69 and an RMSE value of 0.51 µmol CO_2_ m^−2^ s^−1^ (Fig. 8). The tendency of kriging to overestimate small values is supported by previous studies [80]–[82]. This tendency may help explain the bias toward overestimating *R_s_* at low values (Fig. 8). Therefore, improving the accuracy of input parameters from remote sensing or GIS will increase the predictive capability of the *R_s_* model.

Notably, the *R_s_* model developed in this study was applicable to maize fields during the peak growth period in the three counties in North China. However, the model employed in this study does not consider temperature, a main driver of *R_s_* that has high spatial variability. This model may be not used anywhere else or in other stages of the growing season. Furthermore, when spatially distributed data were used in the *R_s_* model, a simple alternative method was employed to estimate the maize LAI by using the remotely sensed EVI, which may be problematic. Verstraeten et al. [83] highlighted that the assimilation of remotely sensed geophysical products into a carbon model is a complex process, and simply exchanging conventional input data for their remotely sensed counterparts is insufficient. Therefore, future research should focus on an integrating spatially distributed *R_s_* datasets and geophysical products from remote sensing and GIS by using the data assimilation method, which has been extensively applied in terrestrial carbon cycle research [84]–[86]. However, this method lack the integration of *R_s_* and spatially distributed data.

## Conclusions

This study investigated the potential of spatial data from remote sensing and GIS for estimating the spatial patterns of *R_s_* during the peak growing season of maize in three counties in North China. Based on in situ measurements, plant productivity (i.e., LAI) and soil property (i.e. SOC content) factors were identified as the most important determinants of spatial variability in *R_s_* during the peak growing season of maize, and *R_s_* was weakly related to soil temperature and soil moisture. Spectral VIs calculated from an HJ-1A CCD optical image were used to estimate LAI and EVI was found to be the best proxy for LAI. To derive the spatial pattern of *R_s_* during the peak growing season of maize, a simple exponential model, which included remotely sensed EVI and GIS spatially interpolated SOC content, was employed. This method was tested by using an independent sample dataset and was shown to be reasonable at the study site.
